# Sarcopenia is associated with the presence of nonalcoholic fatty liver disease in Zhejiang Province, China: a cross-sectional observational study

**DOI:** 10.1186/s12877-020-01910-3

**Published:** 2021-01-14

**Authors:** Yu-Ming Wang, Ke-Fu Zhu, Wen-Jing Zhou, Qin Zhang, Dan-Feng Deng, Yi-Chen Yang, Wen-Wen Lu, Jia Xu, Yun-Mei Yang

**Affiliations:** 1 Department of Geriatrics, the First Affiliated Hospital, School of Medicine, Zhejiang University, Hangzhou, 310003 Zhejiang China; 2 Zhejiang Provincial Key Laboratory for Diagnosis and Treatment of Aging and Physic-chemical Injury Diseases, the First Affiliated Hospital, School of Medicine, Zhejiang University, Hangzhou, 310003 Zhejiang China; 3 Department of Cardiology, Zhejiang Hospital, Hangzhou, 310013 Zhejiang China; 4 Department of Emergency Medicine, The First Affiliated Hospital, School of Medicine, Zhejiang University, Hangzhou, 310003 Zhejiang China

**Keywords:** Muscle mass, Muscle strength, Non-alcoholic fatty liver disease, Sarcopenia

## Abstract

**Background:**

Currently, both non-alcoholic fatty liver disease (NAFLD) and sarcopenia have attracted extensive attention in public health. However, the relationship between NAFLD and sarcopenia remains unclear. This study aimed to clarify the sex-specific association between sarcopenia and NAFLD according to the Asian Working Group for Sarcopenia (AWGS).

**Methods:**

Dual-energy X-ray absorptiometry (DXA) and hepatic ultrasonography were measured in 578 participants (92 men and 486 women) during their annual health examinations. Multivariate logistic regression models were used to explore the association between NAFLD and sarcopenia with its two components.

**Results:**

A total of 154 participants (30 men and 124 women) had NAFLD. The prevalence of sarcopenia was higher among the participants with NAFLD than among those without NAFLD (men: 20.0% vs. 9.7%, *P* = 0.295, women: 15.3% vs. 8.0%, *P* = 0.019). Low muscle mass (LMM) was independently associated with NAFLD in both men and women (men: odds ratio [OR], 2.88; 95% confidence interval [CI] 1.52–5.46; women: OR, 2.08; 95% CI 1.63–2.67). However, low muscle strength (LMS) was independently associated with NAFLD only in male participants, with an OR of 1.15 (95% CI 1.02–1.28).

**Conclusion:**

The occurrence of sarcopenia was associated with a higher risk of NAFLD, especially in men, as demonstrated by lower muscle mass and lower muscle strength.

## Background

Non-alcoholic fatty liver disease (NAFLD) is currently the leading cause of chronic liver disease worldwide, ranging from simple steatosis, non-alcoholic steatohepatitis (NASH), and advanced fibrosis to end-stage liver diseases, such as cirrhosis and hepatocellular carcinoma [1]. The total NAFLD population in 2015 was estimated at 83.1 million cases with a prevalence rate of 30.0% among the population aged ≥15 years and will increase to 33.5% by 2030 [2]. NAFLD has been proven to be closely associated with systemic diseases and has attracted extensive attention in public health [3]. The biological mechanisms, such as peripheral resistance to insulin, dyslipidaemia, and the activation of inflammatory pathways associated with NAFLD, are relevant to these systemic diseases [4].

Sarcopenia is a multifactorial geriatric syndrome with the overall concept of skeletal muscle failure or insufficiency. Skeletal muscle, as an active endocrine organ responsible for insulin-mediated glucose disposal, plays a significant role in glucose homeostasis, insulin resistance and inflammation [5]. Meanwhile, sarcopenia is associated with a sedentary lifestyle, which increases the risk of obesity, metabolic syndrome and NAFLD [6]. It has been reported that up to 60% of patients with end-stage liver disease have sarcopenia. The presence of NASH in those patients was associated with a 6-fold increased risk of sarcopenic obesity [7, 8]. Thus, sarcopenia shares the common risk factors that contribute to NAFLD and have a plausible association with NAFLD.

Until now, all consensuses have agreed on two crucial components of the sarcopenia definition: sarcopenia involves both structural damage (low muscle mass [LMM]) and impaired function (low muscle strength [LMS]) [9]. Several studies have shown that sarcopenia, including its two crucial components (LMM and LMS), is associated with the prevalence of NAFLD. Kim et al. found that skeletal muscle mass was positively correlated with the occurrence of NAFLD and negatively correlated with the resolution of existing NAFLD [10]. Another study showed that men and women with NAFLD had 7.3 and 7.9% lower handgrip strength (HGS) than controls in older adults [11]. Since the trajectories of muscle mass and muscle strength decline during ageing do not overlap and muscle strength declines much more rapidly than muscle mass, it is essential to illustrate the association between NAFLD and sarcopenia with its two components at the same time [12, 13]. Furthermore, almost all clinical studies were conducted in Korean populations, and few were diagnosed according to the Asian Working Group for Sarcopenia (AWGS) consensus guidelines. Therefore, this study aimed to investigate the independent association of the two components of sarcopenia with NAFLD, stratified by sex, in the aged Chinese population according to the AWGS. In addition, we tried to assess which component could better predict NAFLD prevalence in different sex groups.

## Methods

### Study population

This is a cross-sectional, observational study of 578 senior hospital staff (92 men and 486 women) who attended annual health examinations at the First Affiliated Hospital, School of Medicine, Zhejiang University, between January 2019 and December 2019. Participants who had full records of personal health history, anthropometric and biochemical data and hepatic ultrasonography results were initially enrolled. Participants who had cancer, viral/drug-induced/autoimmune liver diseases, severe cardiopulmonary disorders, renal dysfunction, history of organ transplant, physical or cognitive impairment, or excessive alcohol consumption (males > 140 g/week or females > 70 g/week) were excluded [14]. This study was approved by the Ethics Committee of The First Affiliated Hospital, School of Medicine, Zhejiang University, in accordance with the Helsinki Declaration. All participants gave written informed consent before participation.

### Laboratory measurements

All participants underwent a thorough physical examination. Height (cm) and weight (kg) were measured using standardized protocols while the participants were dressed in light clothing without shoes. Body mass index (BMI) (kg/m^2^) was calculated according to the following formula: BMI = weight (kg)/height (m)^2^. According to Asia-Pacific criteria, general obesity was defined as BMI ≥ 25 kg/m^2^. After an overnight fast of ≥8 h, blood samples were obtained from the peripheral vein of each participant. All laboratory measurements, including liver function tests (alanine aminotransferase [ALT], aspartate aminotransferase [AST], and gamma-glutamyltransferase [GGT]), lipid profile (triglyceride [TG], total cholesterol [TC], high-density lipoprotein cholesterol [HDL-C], and low-density lipoprotein cholesterol [LDL-C]), fasting plasma glucose (FPG), albumin (ALB), creatinine (Cr) and uric acid (UA), were measured by a Hitachi 7600 autoanalyser (Hitachi, Tokyo, Japan) using standard protocols.

### Diagnosis of sarcopenia and relative parameters

**Fig. 1:**
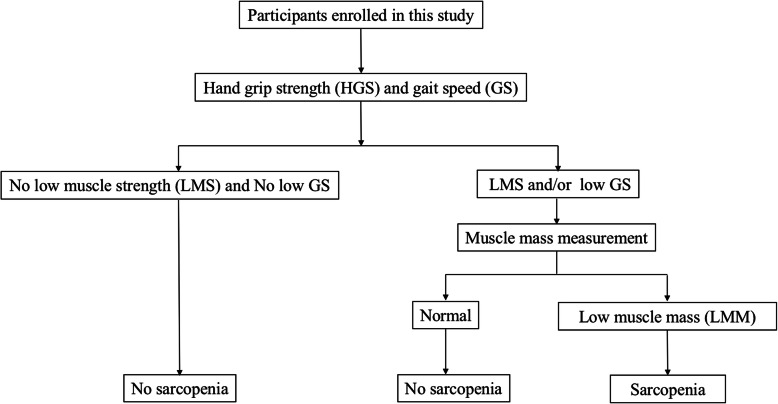
Recommended diagnostic algorithm of Asian Working Group for Sarcopenia (AWGS)

Participants who had completed dual-energy X-ray absorptiometry (DXA) tests for HGS and 4 m walking speed were selected. A gait speed (GS) < 0.8 m/s was defined as low GS, as recommended by the AWGS. Both the left and right HGSs were measured with a Jamar Hydraulic Hand Dynamometer (Jamar Hydraulic Hand Dynamometer Model 5030 J1; Sammons Preston, Bolingbrook, IL, USA) three times, and the maximum value was used, according to the recommendations of the American Society of Hand Therapists (ASHT). LMS was defined as < 26.0 kg in men and < 18.0 kg in women, as recommended by the AWGS. Skeletal muscle mass was estimated by the skeletal muscle index (SMI) using the appendicular skeletal muscle mass (ASM) divided by body height squared (kg/m^2^), which was measured by DXA using a Hologic Discovery™ device (Hologic, Waltham, MA, USA). LMM was defined as ≤7.0 kg/m^2^ in men and ≤ 5.4 kg/m^2^ in women as recommended by the AWGS. Sarcopenia was diagnosed according to the criteria of the AWGS [15] (Fig. 1). Sarcopenic obesity was defined as the presence of both sarcopenia and obesity.

### Diagnosis of NAFLD

NAFLD was determined based on the result of a hepatic ultrasound examination following the exclusion of alcohol consumption and viral or autoimmune liver disease. Hepatic ultrasonography (US; Acuson Sequoia 512, Siemens, Mountain View, CA, USA) was carried out by experienced ultrasonographers. The ultrasonographers were blinded to study design and clinical data. The criteria for the ultrasonic diagnosis of fatty liver were based on those recommended by the Chinese Liver Disease Association [16].

### Statistical analysis

Continuous variables are presented as the mean ± standard deviation (SD), and categorical variables are presented as the frequency (percentage). The statistical significance of the differences in clinical and biochemical values between participants with and without NAFLD was analysed by sex using Student’s t-test for continuous variables and the chi-squared test for categorical variables. The Pearson correlation coefficient was calculated to assess the associations between muscle mass or muscle strength and the relative parameters of NAFLD. Multivariate logistic regression models were performed to calculate the adjusted OR and 95% CI to explore the associations of NAFLD with muscle mass and muscle strength. The following factors were considered independent variables for multivariate logistic regression analysis: Model 1: age and weight; Model 2: model 1 covariates plus BMI, TG, and ALT. A receiver operating characteristic (ROC) curve of muscle mass was developed to predict the presence of NAFLD in both men and women. All calculations were performed using IBM SPSS Statistics ver. 22.0 (IBM Co., Armonk, NY, USA), and the associated results were plotted using GraphPad Prism 6 (GraphPad, San Diego, California, USA). Two-sided *P*-values < 0.05 were considered statistically significant.

## Result

### Basic laboratory and clinical characteristics

The basic laboratory and clinical characteristics of the 578 participants (92 men and 486 women) with and without NAFLD enrolled in this study are shown in Table 1. Thirty (32.6%) male and 124 (25.5%) female participants had NAFLD. Among the men, the participants with NAFLD were younger and had higher body weight, BMI, serum TG, and ALT than those without NAFLD, but there were no differences in height, serum TC, HDL-C, LDL-C, AST, GGT, ALB, Cr, FPG or UA between the two groups. Among the women, the participants with NAFLD were older and had higher weight, BMI, TG, serum AST, ALT, GGT, ALB, FPG, and UA and lower HDL-C than those without NAFLD. However, there were no differences in height, serum TC, LDL-C or Cr between the two groups. As illustrated in Table 1, the levels of GS were lower in patients with NAFLD than in NAFLD-free participants in both men and women (men: 1.1 ± 0.3 vs. 1.3 ± 0.3 kg/m^2^, *P* = 0.008; women: 1.1 ± 0.3 vs. 1.2 ± 0.2 kg/m^2^, *P* = 0.008). The levels of muscle mass were also significantly lower in patients with NAFLD than in NAFLD-free participants (men: 6.6 ± 1.2 vs. 7.2 ± 1.3 kg/m^2^, *P* = 0.032; women: 5.2 ± 1.3 vs. 5.6 ± 1.1 kg/m^2^, *P* = 0.011). However, there was no significant difference in the levels of HGS between the two groups (men: 35.8 ± 8.6 vs. 37.6 ± 9.7, *P* = 0.404, women: 23.4 ± 5.8 vs. 24.5 ± 5.1 kg, *P* = 0.053). The prevalence of sarcopenia was higher among the participants with NAFLD (men: 20.0% vs. 9.7%, *P* = 0.295, women: 15.3% vs. 8.0%, *P* = 0.019). Moreover, the prevalence of sarcopenic obesity was also higher in the NAFLD group in both men and women, but the difference was not significant (men: 10.0% vs. 3.2%, *P* = 0.394, women: 3.2% vs. 0.8%, *P* = 0.134).

**Table 1:** Baseline characteristics of the study participants, categorized according to the presence or absence of NAFLD

	Men(***n*** = 92)			Women(***n*** = 486)		
Variable	Without NAFLD (***n*** = 62)	With NAFLD (***n*** = 30)	***P***	Without NAFLD (***n*** = 362)	With NAFLD (***n*** = 124)	***P***
Ages (years)	**72.9 ± 9.2**	**68.9 ± 5.8**	**0.029**	**62.9 ± 12.1**	**67.5 ± 10.1**	**<0.001**
Weight (kg)	**67.3 ± 9.6**	**75.3 ± 12.2**	**<0.001**	**55.8 ± 7.0**	**61.5 ± 8.3**	**<0.001**
Height (cm)	170.7 ± 6.1	169.0 ± 6.4	0.239	157.3 ± 9.4	157.7 ± 4.9	0.587
Body mass index (kg/m^2^)	**23.6 ± 3.0**	**25.7 ± 3.3**	**0.002**	**22.5 ± 2.6**	**24.7 ± 2.8**	**<0.001**
TC (mmol/L)	4.4 ± 0.9	4.6 ± 0.9	0.312	5.0 ± 0.9	5.1 ± 1.1	0.711
TG (mmol/L)	**0.8 ± 0.5**	**1.8 ± 0.7**	**<0.001**	**1.2 ± 0.7**	**1.6 ± 1.0**	**<0.001**
HDL-C (mmol/L)	1.3 ± 0.3	1.2 ± 0.4	0.065	**1.5 ± 0.4**	**1.3 ± 0.3**	**<0.001**
LDL-C (mmol/L)	2.5 ± 0.7	2.6 ± 0.8	0.426	2.8 ± 0.7	2.9 ± 0.9	0.321
AST (IU/L)	23.0 ± 3.1	23.6 ± 4.4	0.665	**21.3 ± 6.0**	**26.1 ± 7.2**	**0.003**
ALT (IU/L)	**19.3 ± 7.4**	**25.5 ± 14.0**	**0.007**	**17.6 ± 10.2**	**24.5 ± 14.6**	**<0.001**
GGT (IU/L	28.0 ± 17.6	29.5 ± 13.7	0.696	**21.2 ± 16.8**	**28.4 ± 18.4**	**<0.001**
ALB (g/L)	46.6 ± 2.4	47.6 ± 2.0	0.061	**46.9 ± 2.4**	**47.9 ± 2.5**	**<0.001**
Cr (umol/L)	89.1 ± 15.3	87.2 ± 12.3	0.555	64.9 ± 11.0	64.6 ± 11.1	0.789
FPG (mmol/L)	5.6 ± 1.4	5.9 ± 1.1	0.236	**5.2 ± 1.0**	**5.8 ± 1.2**	**<0.001**
UA (μmol/L)	357.5 ± 84.2	371.0 ± 77.5	0.461	**267.3 ± 63.4**	**305.4 ± 71.0**	**<0.001**
Gait speed (m/s)	**1.3 ± 0.3**	**1.1 ± 0.3**	**0.008**	**1.2 ± 0.2**	**1.1 ± 0.3**	**0.008**
Muscle strength (kg)	37.6 ± 9.7	35.8 ± 8.6	0.404	24.5 ± 5.1	23.4 ± 5.8	0.053
Muscle mass (kg /m^2^)	**7.2 ± 1.3**	**6.6 ± 1.2**	**0.032**	**5.6 ± 1.1**	**5.23 ± 1.3**	**0.011**
Sarcopenia	6/62 (9.7%)	6/30 (20.0%)	0.295	**29/362 (8.0%)**	**19/124 (15.3%)**	**0.019**
Sarcopenic obesity	2/62 (3.2%)	3/30 (10.0%)	0.394	3/362 (0.8%)	4/124 (3.2%)	0.134

Values are presented as mean ± standard deviation, unless otherwise specified. Bold numbers indicate statistically significant values. *NAFLD* non-alcoholic fatty liver disease, *BMI* body mass index, *TC* total cholesterol, *TG* triglyceride, *HDL-C* high-density lipoprotein cholesterol, *LDL-C* low-density lipoprotein cholesterol, *AST* aspartate aminotransferase, *ALT* alanine aminotransferase, *GGT* gamma-glutamyl transferase, *ALB* albumin, *Cr* creatinine, *FPG* fasting plasma glucose, *UA* uric acid

### Associations of muscle mass and anthropometric and biochemical variables of NAFLD

We performed Pearson correlation analysis to determine the correlations between muscle mass and anthropometric and biochemical variables of NAFLD. The correlation analyses between anthropometric and biochemical variables and muscle mass in participants are shown in Table 2. We found that muscle mass was positively correlated with body weight (*r* = 0.292, *P* = 0.005), BMI (*r* = 0.291, *P* = 0.005), ALB (*r* = 0.232, *P* = 0.026) and HGS (*r* = 0.315, *P* = 0.002) but negatively correlated with age (*r* = − 0.244, *P* = 0.019) and FPG (*r* = − 0.251, *P* = 0.016) among male participants. Meanwhile, muscle mass was positively correlated with age (*r* = 0.111, *P* = 0.015), body weight (*r* = 0.295, *P*<0.001), BMI (*r* = 0.326, *P*<0.001), serum ALT (*r* = 0.139, *P* = 0.002), Cr (*r* = 0.193, *P*<0.001), UA (*r* = 0.142, *P* = 0.002), and FPG (*r* = 0.111, *P* = 0.015) among female participants (Table 2).

**Table 2:** Pearson correlation coefficients between muscle mass and patient characteristics at baseline by gender (for men and women)

Variable	Muscle mass (kg/m^2^)			
	men		women	
	r	*P*	r	*P*
Age (years)	− 0.244	0.019	0.111	0.015
Body weight (kg)	0.292	0.005	0.295	<0.001
Height (cm)	0.098	0.355	−0.078	0.088
BMI (kg/m^2^)	0.291	0.005	0.326	<0.001
ALT (IU/L)	0.093	0.378	0.139	0.002
AST (IU/L)	0.243	0.167	0.177	0.101
TG (mmol/L)	−0.025	0.815	−0.01	0.834
TC (mmol/L)	−0.108	0.304	−0.055	0.232
ALB (g/L)	0.232	0.026	−0.003	0.943
Cr (μmol/L)	0.101	0.339	0.193	<0.001
UA (μmol/L)	0.179	0.087	0.142	0.002
FPG (mmol/L)	−0.251	0.016	0.111	0.015
Gait speed (m/s)	0.153	0.145	−0.026	0.568
Muscle strength (kg)	0.315	0.002	−0.041	0.364

### Associations of HGS and anthropometric and biochemical variables of NAFLD

We found that HGS was positively correlated with body weight (*r* = 0.255, *P* = 0.014), height (*r* = 0.51, *P*<0.001), GS (*r* = 0.408, *P*<0.001) and muscle mass (*r* = 0.315, *P* = 0.002) but negatively correlated with age (*r* = − 0.504, *P*<0.001) among male participants. HGS was positively correlated with body weight (*r* = 0.206, *P*<0.001), GS (*r* = 0.538, *P*<0.001) and height (*r* = 0.315, *P*<0.001) but negatively correlated with age (*r* = − 0.533, *P*<0.001), serum AST (*r* = − 0.391, *P*<0.001), TG (*r* = − 0.144, *P* = 0.002), UA (*r* = − 0.193, *P*<0.001), and FPG (*r* = − 0.177, *P*<0.001) among female participants (Table 3).

**Table 3:** Pearson correlation coefficients between muscle strength and patient characteristics at baseline by gender (for men and women)

Variable	Muscle strength (kg)			
	men		women	
	r	*P*	r	*P*
Age (years)	−0.504	<0.001	−0.533	<0.001
Body weight (kg)	0.255	0.014	0.206	<0.001
Height (cm)	0.51	<0.001	0.315	<0.001
BMI (kg/m^2^)	0.02	0.848	−0.018	0.692
ALT (IU/L)	0.062	0.554	−0.065	0.156
AST (IU/L)	0.08	0.651	−0.391	<0.001
TG (mmol/L)	0.15	0.152	−0.144	0.002
TC (mmol/L)	0.019	0.854	0.011	0.804
ALB (g/L)	0.184	0.079	0.142	0.002
Cr (μmol/L)	0.013	0.901	−0.155	0.001
UA (μmol/L)	0.059	0.578	−0.193	<0.001
FPG (mmol/L)	−0.195	0.062	−0.177	<0.001
Gait speed (m/s)	0.408	<0.001	0.538	<0.001
Muscle mass (kg/m^2^)	0.315	0.002	−0.041	0.364

### Independent impact of muscle mass and muscle strength on the presence of NAFLD

A logistic regression model was conducted to evaluate the sex-specific relationship between the components of sarcopenia (LMM or LMS) and NAFLD risk (Models 1–2, Table 4). The relationship between LMM and NAFLD was statistically significant in both models. In Model 1, the ORs with 95% CIs for NAFLD were 2.91 (95% CI 1.58–5.35) and 1.89 (95% CI 1.51–2.38) in men and women, respectively. Furthermore, the fully adjusted model (Model 2) showed that LMM was still associated with an increased risk of NAFLD, with ORs of 2.88 (95% CI 1.52–5.46) in men and 2.08 (95% CI 1.63–2.67) in women. Participants with LMS showed significantly high odds of NAFLD, with ORs of 1.15 (95% CI, 1.04–1.26) and 1.15 (95% CI 1.02–1.28) in men after adjusting in Model 1 and Model 2, respectively. However, there was no statistical significance in muscle strength in women after adjusting in Model 1 and Model 2, with ORs of 1.05 (95% CI 0.99–1.11) and 1.01 (95% CI 0.95–1.07), respectively.

**Table 4:** ORs and 95% CIs of muscle mass and muscle strength for NAFLD

	Men			
Variable	Model 1		Model 2	
	OR (95%CI)	*P*	OR (95%CI)	*P*
Muscle mass, per SD decrease	**2.91 (1.58, 5.35)**	**0.001**	**2.88 (1.52, 5.46)**	**0.001**
Muscle strength, per SD decrease	**1.15 (1.04, 1.26)**	**0.004**	**1.15 (1.02, 1.28)**	**0.021**
	Women			
Variable	Model 1		Model 2	
	OR (95%CI)	*P*	OR (95%CI)	*P*
Muscle mass, per SD decrease	**1.89 (1.51, 2.38)**	**<0.001**	**2.08 (1.63, 2.67)**	**<0.001**
Muscle strength, per SD decrease	1.05 (0.99, 1.11)	0.058	1.01 (0.95, 1.07)	0.716

Results are given as OR (95% CI) for NAFLD as outcome stratified by gender. Results in bold reflect significant findings with a *P* value < 0.05. Model 1: adjusted for age and weight. Model 2: adjusted for age, weight, BMI, TG, ALT. NAFLD, nonalcoholic fatty liver disease; BMI, body mass index; TG, triglyceride; ALT, alanine aminotransferase

### ROC curve of muscle mass

**Fig. 2:**
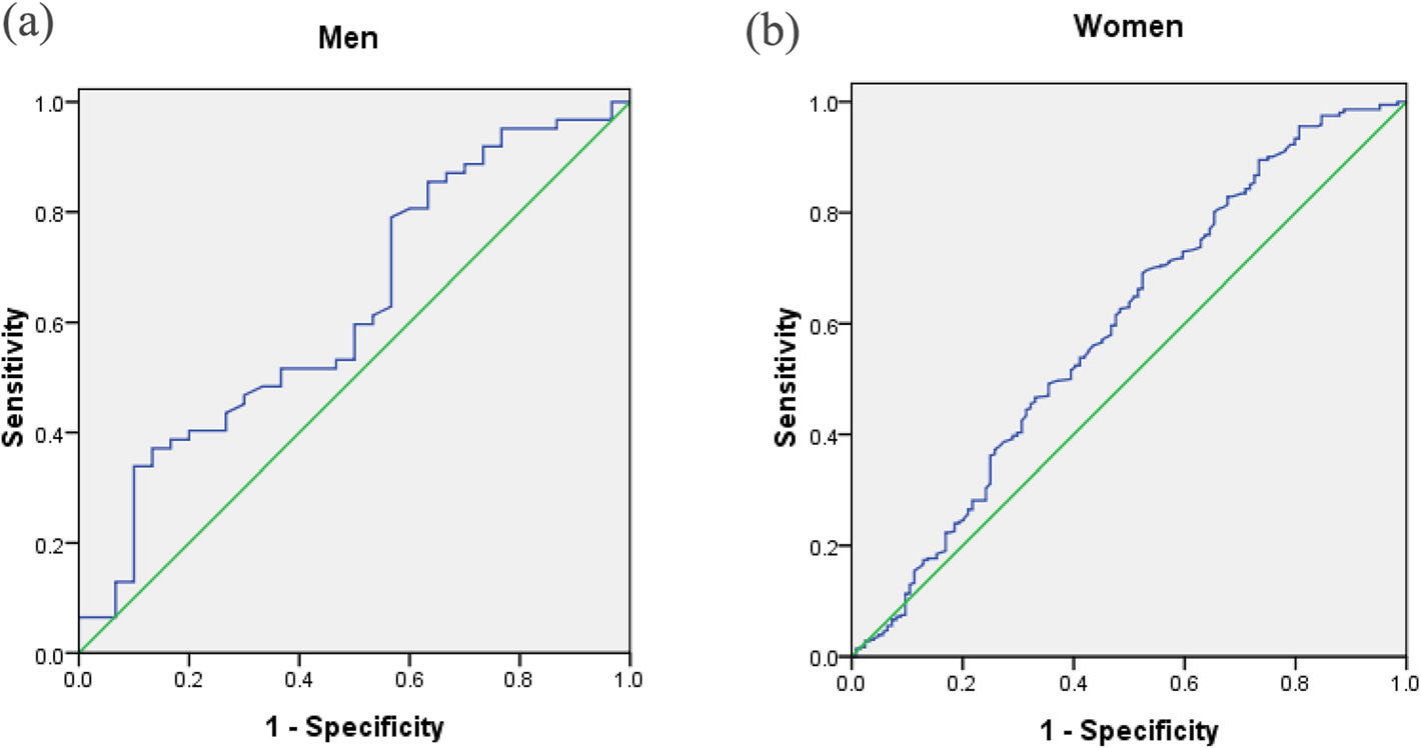
ROC curves of muscle mass to predict the presence of NAFLD by sex. **a** Men (AUC = 62.4%, *P* < 0.063); **b** Women (AUC = 59.2%, *P* < 0.031)

The ROC curves of muscle mass plotted for the diagnosis of NAFLD by sex are shown in Fig. 2. The cut-off value of muscle mass was 8.0 kg/m^2^ in men and 4.9 kg/m^2^ in women, with sensitivities of 33.9 and 68.8% and specificities of 90.0 and 47.6%, respectively. The areas under the ROC curve for NAFLD were 0.624 (95% CI 0.501–0.748, *P* < 0.063) and 0.592 (95% CI 0.531–0.653, *P* < 0.031) in men and women, respectively.

## Discussion

NAFLD is a spectrum of liver disease that has now become the most common cause of chronic liver disease in adults of all ethnicities. The risk of NAFLD increases with age [17]. Sarcopenia is described as involuntary loss of muscle mass, muscle strength and muscle performance that occurs with ageing, which can cause adverse health outcomes such as falls, disability, poor quality of life, and even mortality according to the AWGS. The AWGS also recommends cut-off values for muscle mass measurements (7.0 kg/m^2^ for men and 5.4 kg/m^2^ for women by using DXA), HGS (< 26 kg for men and < 18 kg for women), and usual gait speed (< 0.8 m/s). Sarcopenia is considered a new geriatric syndrome that is closely associated with metabolic disorders, which denotes its importance in health care for older people [18]. In this cross-sectional sample of a Chinese study, we aimed to examine the sex-specific association between NAFLD and sarcopenia with its two crucial components using hepatic imaging and DXA scans, according to the AWGS criteria. Our data indicated that men and women with NAFLD both had markedly lower muscle mass and were more likely to have lower muscle strength than controls. The status of sarcopenia and sarcopenic obesity leads to an increased prevalence of NAFLD. Moreover, LMM appeared to be a better predictor for NAFLD prevalence than LMS. Further multivariable analysis identified that participants with LMM had statistically higher odds of suffering from NAFLD than participants with LMS in both men and women.

Recent clinical studies have already demonstrated a positive relationship between sarcopenia and the prevalence of NAFLD [6, 11, 19, 20]. Hong et al. first found that the OR for NAFLD risk was 5.16 (95% CI 1.63–16.33) in the lowest quartile of SMI compared to the highest quartile [6]. Kim et al. showed that men and women with NAFLD had markedly lower HGS and were more likely to have LMS than controls [11]. However, most of these studies focused on a single component of sarcopenia, which was not sufficient to fully understand the relationship between muscle status and NAFLD. It is unclear whether LMM or LMS is independently associated with NAFLD in the same model. In the present study, we found that men and women with NAFLD had markedly lower SMIs and were more likely to have LMM than controls. LMM was associated with an increased risk of NAFLD, with ORs of 2.88 (95% CI 1.52–5.46) in men and 2.08 (95% CI 1.63–2.67) in women. However, participants with LMS showed a slightly higher odds of NAFLD, with an OR of 1.15 (95% CI 1.02–1.28) in men only.

Moreover, since sarcopenia has been described as an age-associated decline in muscle mass as well as muscle function (defined by muscle strength or physical performance), it is also important to focus on the relationship between physical performance and NAFLD. We found that subjects with NAFLD had significantly lower GS than NAFLD-free subjects in both men and women. Further Pearson correlation coefficients showed that GS had a positive association with muscle strength (men: *r* = 0.315, *P* = 0.002, women: *r* = 0.538, *P*<0.001). A wide range of tests for physical performance are recommended when diagnosing sarcopenia, including the Short Physical Performance Battery (SPPB), usual gait speed walk test, stair climb power test, and timed-up-and-go test (TUG) [15]. A Taiwanese study examined the association of NASH with physical fitness, which demonstrated that men with NASH had fewer 2-min sit-up numbers and longer times to complete a 3000-m run than unaffected men [21]. Exercise, such as aerobic exercise, resistance training, and their combination, can increase muscle protein synthesis and promote anabolism, which can effectively improve the progression of sarcopenia and NAFLD [22, 23]. Therefore, since both NAFLD and sarcopenia may result in reduced physical capability and poor quality of life, the status of comorbidity in older adults should be given extensive attention.

Previous studies also attempted to elucidate the mechanism of sarcopenia development in patients with NAFLD. Currently, growing attention has been given to sex-specific differences in the development of sarcopenia and NAFLD. In this study, men with LMM had an increased risk of suffering from NAFLD, with higher odds after adjustment compared to female participants. Regarding muscle strength, low HGS was associated with an increased NAFLD prevalence in men only. Yang et al. found that some metabolic syndromes may make men more prone to sarcopenia, likely due to the low levels of physical activity associated with such conditions [24]. A small cross-sectional study from Japan revealed that the SMI had a negative association with hepatic steatosis only in men with type 2 diabetes [25]. Then, a more extensive population-based study involving 4210 men and women also suggested that sarcopenia was independently associated with NAFLD in men with type 2 diabetes, while no significant difference was found in women [26]. In our study, we found that male subjects with NAFLD were younger than those without NAFLD; in contrast, female subjects with NAFLD were older than those without NAFLD. In addition to the reasons of genetic predisposition and different lifestyles, changes in basal hormone levels with age may also lead to this opposite outcome between older men and women. Some studies have confirmed that dysregulated sex hormone disorders are involved in the pathogenesis of NAFLD and sarcopenia. Testosterone, a potent anabolic hormone, can promote muscle protein synthesis [27]. Sarcopenia, which is related to the reduction in physical activity, the lack of anabolic hormones, and the decrease in proinflammatory cytokines, has been associated with NAFLD independent of metabolic syndrome features [5, 28]. Extrapolating from these findings, the regulation of sex hormones may be involved in the mechanism of sex-specific differences in the development of sarcopenia and NAFLD.

Several epidemiological and experimental studies have shown that insulin resistance may have an essential role in sarcopenia. Skeletal muscle is recognized as a tissue that is primarily responsible for peripheral insulin-mediated glucose disposal. Insulin resistance may reduce the stimulation of the protein synthesis pathway and increase the activation of the protein degradation pathway, which might eventually lead to muscle loss [18, 29]. Indeed, the prevalence of sarcopenic obesity was higher among the participants with NAFLD in our study. Meanwhile, Pearson correlation coefficient analyses confirmed that the level of FPG was inversely associated with muscle mass in men and HGS in women. The serum level of TG was also negatively correlated with HGS in women. In this context, our current research also implied that both LMM and LMS might participate in the progression of NAFLD through dyslipidaemia and insulin resistance.

It is also worth mentioning that compared to LMS, LMM had higher odds of increasing the risk of NAFLD in our study. In women, sarcopenia was only found to have a statistically significant relationship with NAFLD when defined in terms of muscle mass alone, which was consistent with early research focused on the relationship between sarcopenia and metabolic syndrome. This might suggest that muscle mass, to some extent, was more significant than muscle strength in the context of NAFLD.

However, several limitations should also be considered when interpreting the results. First, the diagnosis of NAFLD was based on hepatic ultrasonography, and histologic confirmation of NAFLD by liver biopsy was not available. Second, the study was limited to verifying causality due to the cross-sectional design. Third, the information regarding past medical history was self-reported, which might have led to recall bias. Additionally, the number of subjects with sarcopenic obesity was low, which needs further enlargement of the sample size to allow for an adequate statistical comparison. Finally, our study population was exclusively Chinese, so the results might not be generalizable to other populations.

## Conclusion

This work is among the few studies to examine the independent association of the two crucial components of sarcopenia (LMM and LMS) with NAFLD, stratified by sex according to the AWGS. LMM was consistently associated with NAFLD in both men and women, while LMS was associated with NAFLD only in men after adjustment for potential confounders. Moreover, compared to muscle strength, muscle mass was a better predictor for the presence of NAFLD in both sexes. Given that the understanding of the close relationship between NAFLD and sarcopenia is of great interest in this era of the ageing population, further well-designed studies should focus on common therapeutic strategies to prevent muscle wasting as well as NAFLD.

## Data Availability

The datasets used and/or analysed during the current study are available from the corresponding author on reasonable request.

## Abbreviations

ALB: Albumin
ALT: Alanine aminotransferase
ASM: Appendicular skeletal muscle mass
AST: Aspartate aminotransferase
AWGS: Asian Working Group for Sarcopenia
CI: Confidence interval
Cr: Creatinine
DXA: Dual-energy X-ray absorptiometry
FPG: Fasting plasma glucose
GGT: Gamma-glutamyltransferase
GS: Gait speed
HDL-C: high-density lipoprotein cholesterol
HGS: Handgrip strength
LDL-C: Low-density lipoprotein cholesterol
LMM: Low muscle mass
LMS: Low muscle strength
NAFLD: Non-alcoholic fatty liver disease
NASH: Non-alcoholic steatohepatitis
OR: Odds ratio
SMI: Skeletal muscle index
TC: Total cholesterol
TG: Triglyceride
